# Self-medication during pregnancy and associated factors among pregnant women in Goba town, southeast Ethiopia: a community based cross sectional study

**DOI:** 10.1186/s13104-018-3821-8

**Published:** 2018-10-10

**Authors:** Taye Zewdie, Telake Azale, Alemayehu Shimeka, Ayenew Molla Lakew

**Affiliations:** 1 Department of Nursing, Alkan University College Goba, Addis Ababa, Ethiopia; 2 Department of Health Education and Behavioral Science, College of Medicine and Health Sciences, University of Gondar, Gondar, Ethiopia; 3 Department of Epidemiology and Biostatistics, College of Medicine and Health Sciences, University of Gondar, Gondar, Ethiopia

**Keywords:** Self-medication, Pregnant women, Southeast Ethiopia

## Abstract

**Objective:**

The use of self-medications during pregnancy results in serious structural as well as functional adverse effects on mothers and unborn children. But little is known about the practice of self-medication used during pregnancy in Ethiopia. Therefore, this research aimed to assess the prevalence of self-medication practice and associated factors during pregnancy among pregnant women in Goba town, southeast Ethiopia.

**Results:**

The prevalence of self-medication was 15.5% (95% CI 0.116, 0.195) in Goba town. Women who had health problems during pregnancy (AOR = 6.1, 95% CI 2.67, 13.9), women unable to read and write (AOR = 8.87, 95% CI 1.84, 41.95), those who can read and write (AOR = 5.26, 95% CI 1.34, 20.66) and had primary education (AOR = 3.57, 95% CI 1.42, 9.02) were more likely to use self-medication, while women who visited ANC for pregnancy (AOR = 0.028, 95% CI 0.09, 0.87) were less likely to indulge on such practices. In conclusion, the prevalence of self-medication noted in this work is medium compared to the react of other studies. Health institutions have to give health education to all pregnant women attending ANC services regardless of gestational age and types of health problem.

**Electronic supplementary material:**

The online version of this article (10.1186/s13104-018-3821-8) contains supplementary material, which is available to authorized users.

## Introduction

Self-medication is defined as the use of manufactured or homemade drugs without medical prescriptions seeking to treat symptoms or self-diagnosed health conditions [1, 2]. In many developing countries where drugs are not well-regulated analgesics, anti-malarial, antibiotics, and cough syrups drugs are prone to self-medication of which analgesics are the most commonly used preparations [3, 4].

There is a substantial variation in the prevalence of self-medication among developing and developed countries because of inherent cultural and socioeconomic differences, disparities in health care systems and access to health care plus drug dispensing policies [5]. The practice has been high all over the world, for example, up to 68% in European countries [6]. In developing countries, people, especially pregnant women are using both non-prescription and prescription drugs without supervision [3, 7, 8].

People with low socioeconomic status use medications recommended by relatives who previously used the same medication [9]. The patterns of self-medication vary across populations [10].

Although some of the drugs are intended for self-medication and are of established efficacy and safety, they have serious insinuation, especially in some groups like children, elders, pregnant and lactating mothers due to inappropriate use and lack of knowledge about their side effects and interactions [11, 12]. Pregnancy is a special physiological condition when drug treatment presents needs for a special concern. Inappropriate use of medications during pregnancy may result in serious structural as well as functional adverse effects on the health of the mother and the development of the child. Even though it is not a direct cause of maternal and child mortality, it could lead to abortion and subsequently death [13–16].

Although it has been reported as something common among pregnant women and resulted in a variety of pregnancy related ailments, previous studies were institution-based liable to selection bias. Besides the study areas were quite different from the current one by their economic status. Thus, this study focused on the assessment of the prevalence and factors associated with self-medication among pregnant women, in the area because no such studies has been done so far.

The finding of this study will provide a baseline information for stakeholders who want to work on the reduction of self-medication practices in order to alleviate unnecessary drug effects on mothers and unborn children. This in turn can help in planning an effective program for the control and monitoring of self-medication among pregnant women in the study area. It will also be used as a baseline for further studies.

## Main text

### Methods

A community-based cross-sectional study was conducted in Goba town, southeastern Ethiopia, from April 15 to May 15, 2015. Goba town is located 445 km southeast of Addis Ababa. In 2015, the town had an estimated 1651 pregnant women. The source population comprised all pregnant women living in the town. Pregnant women who were on labor pain, had serious illness, and unable to communicate at the time of data collection were excluded.

A sample of 323 participants was included using the single population formula considering the prevalence of self-medication at Jimma town (20.1%) [17], 95% confidence level, 4% marginal error, and adding a 5% non-response rate.

Participants were selected out of the six administrative kebeles of the town using the simple random sampling technique. From each kebele lists of households with pregnant women were obtained from health extension workers. A sampling frame was designed and participants were proportionally drafted in each selected kebele on the basis of the simple random selection technique. In case of more than one pregnant woman in selected households, one of them was taken by the lottery method.

The dependent variable of this study was self-medication practice (Yes, No). It was defined as “Yes” if a woman used all kinds of pharmaceutical drugs during her pregnancy without medical prescriptions; “No” if she never used or used drugs with therapeutic intent with professional advice/prescriptions. Age, income, occupation, educational level, husband education, parity, gestational age, Health education specific to medication use at ANC session were independent variables considered.

A structured, pre-tested interviewer administered questionnaire translated to the local language, Afaan Oromo, was used. Data was collected by six trained diploma graduate under the supervision of one degree graduate nurse.

The questionnaire was prepared in English and translated into Afaan Oromo, and retranslated to English. A pretest was conducted on 15 pregnant women in Robe town, and amendments were made before the actual data collection. Training was given to data collectors and the supervisor for 3 days. On the spot supervision was also done by the principal investigator and the supervisor.

Data was entered using EpI-INFO Version 3.5.3 and exported to Statistical Package for Social Sciences (SPSS) Version 20.0 for analyses. The frequency distribution was plotted, and logistic regression analysis was used to identify significant independent variables. Then, variables with less than 0.2 p-values in the bi-variable analysis were included in the multivariable logistic regression to identify independent predictors of self-medication practices. Adjusted odds ratio with a 95% confidence interval was used to report the strength of associations. Finally, a significant association was declared at a p-value of less than 0.05.

### Results

#### Socio-demographic characteristics

In this study, 323 pregnant women participated with a response rate of 100%. The mean age of participants was 27 (± SD). Nearly two-thirds, 206 (63.8%), of the respondents were in the age range of 25–34 years. The majority, 160 (49.5%), completed secondary and above classes. About 296 (91.6%) of the women were married, half of them housewives (Table 1).

**Table 1:** Socio-demographic characteristics among pregnant women Goba town south east Ethiopia, 2015

Variables	Frequency	Percent
Age		
15–24	94	29.1
25–34	206	63.8
35 and above	23	7.1
Educational status of women		
Unable to read and write	25	7.7
Read and write	35	10.8
Primary	103	31.9
Secondary and above	160	49.5
Educational status of husband		
Unable to read and write	1	0.3
Read and write	28	8.7
Primary	51	15.8
Secondary	137	42.4
TVET and above	28	8.7
Marital status		
Married	296	91.6
Divorced	12	3.7
Widowed	3	0.9
Unmarried	12	3.7
Ethnicity		
Oromo	210	65.0
Amhara	76	23.5
Somali	8	2.5
Gurage	9	2.8
Other^c^	20	6.2
Religion		
Orthodox	131	40.6
Muslim	152	47.1
Protestant	35	10.8
Catholic	4	1.2
Others^a^	1	0.3
Occupations of the women		
Housewives	160	49.5
GOV Worker	67	20.7
Merchant	59	18.3
Student	8	2.5
Self-employee	28	8.7
Others^b^	1	0.3

*TVET* Technical and vocational educational training

^a^Waqefata

^b^House maid

^c^Sidama, Hareri

#### Obstetric related characteristics

Two-fifths of the women reported that their then pregnancy was the first. The majority, 296 (91.6%), followed ANC services. Seventy (20.1%) of the subjects reported that they experienced health problems during the then pregnancies. The most common self-reported health problems were vomiting and heart burn (Table 2).

**Table 2:** Distribution of pregnancy related conditions and ANC service utilization pattern among pregnant women Goba town south east Ethiopia, 2015

Variables	Categories	Frequency	Percent
Number of pregnancy	One	128	39.6
	Two	116	35.9
	Three and above	79	24.5
Gestational age	First trimester	86	27.0
	Second trimester	118	37.0
	Third trimester	115	36.1
ANC utilization	Yes	296	91.6
	No	24	7.4
Place for ANC visit	Health post	17	5.3
	Health center	167	51.7
	Hospital	126	39.0
	Private clinics	8	2.5
HE on drug utilization at ANC visit	Yes	314	97.2
	No	9	2.8
Faced Health problem during pregnancy	Yes	70	20.1
	No	253	77.7
Type of health problem	Vomiting	13	25
	Back pain	9	17.3
	Headache	6	11.5
	Heart burn	11	21.2
	Constipation	6	11.5
	Cough	3	5.7
	Others^a^	4	7.7

^a^Others includes diahhrea, UTI and abdominal cramp

#### Prevalence of self-medication among pregnant women

Fifty (15.5%, 95% CI 0.116, 0.195) pregnant women used self-medication, about 22 (45%) at least ten times during the pregnancies at the moment. The types of medication commonly used were paracetamol and diclofenac utilized by 17 (29.8%) and 12 (21.0%) participants respectively, and other, like Vermox, Metronidazole, Amoxicillin plus antacids burn were also used.

The main reasons mentioned for self-medication were time saving followed by lack of trust in drugs prescribed by health workers (Additional file 1: Figure S1). The majority, 26 (53.1%), of the respondents got medication from pharmacies, private clinics (20.4), and rural drug vendors, very few (6.1%) from neighbors.

The participants were also asked whether self-medication had effects on infants and women, and all stated that it had no effect. Fifteen (30%) used self-medication during their first trimester, and 40% and 30% used it during their second and third trimesters, respectively.

#### Predictors of self-medication practice

Women’s educational status, ANC follow-up, and health problems were independent predictors of self-medication use (Table 3).

**Table 3:** Logistic regression analysis to identify predictors of self-medication among pregnant women at Goba town, 2015

Variables	COR (95% CI)	AOR (95% CI)
Age of women		
35 and above	1.00	1.00
15–24	1.07 (0.28, 4.12)	0.519 (0.098, 2.75)
25–35	1.32 (0.37, 4.68)	1.25 (0.28, 5.57)
Women educational status		
Secondary and above	1.00	1.00
Unable to read and write	2.21 (0.789, 6.19)	8.78 (1.84, 41.95)
Read and write	0.406 (0.93, 1.17)	5.26 (1.34, 20.66)
Primary	0.799 (3.14, 1.58)	3.57 (1.42, 9.02)
Gestational age		
Third trimester	1.00	1.00
First trimester	0.647 (3.07, 1.41)	1.48 (0.54, 4.09)
Second trimester	0.659 (2.81, 1.36)	1.75 (0.71, 4.33)
Number of pregnancy		
Three and above	1.00	1.00
One	1.63 (0.63, 4.22)	2.98 (0.86, 10.39)
Two	1.95 (0.742, 5.14)	3.34 (0.91, 10.82)
ANC for current pregnancy		
Yes	0.192 (0.081, 0.45)	0.289 (0.10, 0.87)
No	1.00	1.00
Health problem during pregnancy		
Yes	5.21 (2.72, 9.98)	6.12 (2.68, 13.98)
No	1.00	1.00

Pregnant women who were unable to read and write were about nine times more likely to use self-medication compared to secondary school and above graduates. Women who had health problems during pregnancies were over six times more likely to use self-medication their counterparts. The odds of using self-medication were 71% less likely for pregnant women attending ANC services.

### Discussion

In the present study, the prevalence of self-medication was evaluated among pregnant women living in Goba town, southeast Ethiopia. The study revealed that 15.5% of the women used self-medication. The finding is lower than the finding from that of South Africa (59.3%), Palestine (56%), Egypt (86%), and Nigeria (19.6%) [18–21]. This might be due to the variations in socioeconomic status of the countries. It is also lower than the finding of Jimma University Specialized Hospital [17]. The reason may be that the Jimma study was conducted at a hospital, and the women who mostly used health services had health problems and usually used different medications.

However, the prevalence noted in this study is higher than the results in Arak city, Pakistan (12%), Peru (10.5%), Addis Ababa (12.4%) [22–24]. The differences might be due to variations in study settings.

The main reason for using self-medication was time saving for 73.6%. The same reason was given by a study in Jush [25]. Similar to the current finding, the study conducted in Peru showed that time saving and other problems were reported to be the main reasons for self-medication [23]. The reason might be women have different activities at home and outside. They perceived that the operation to take the medicine from health institutions is too long. They also assumed their sickness is related with only pregnancy and they usually used the medicine without consulting health professionals.

In the present study, Paracetamol and Diclofenac were the commonly used medications during pregnancies and a similar finding was published in Peru [23]. This might be due to the accessibility of such drugs with rural drug venders and drug shops.

The most commonly perceived ailments for which pregnant women took self-medication were back pain and heart burns. A study done in Palestine also revealed that pain, heart burn, and indigestion were the commonest ailments that led to self-medications [26]. This might be due to the similarity of pregnancy related ailments.

In the multi-variable analysis, the odds of self-medication use were about nine times higher among women who were unable to read and write, five times among those able to read and write, and three times among who completed primary school compared to those who completed secondary school and above. This finding is similar to a finding from Jush, which revealed that self-medication and maternal education were significantly associated [25]. This may be the fact that women with higher education are more likely to access the media and can get awareness about how medication can be used.

Pregnant women who followed ANC for pregnancies at the moment were 71% less likely to uses self-medication compared to their counterparts. This might be because those who attended ANC got health education/awareness on the uses of drugs during pregnancy and their impacts on mothers and the unborn fetus. The implication is that women were informed and reminded during antenatal classes not to indulge in self-medication to see doctors whenever they had any health concerns no matter how minor it was. It also indicates self-medication can be effectively controlled if women are given proper education about the dangers of taking drugs that are not prescribed by health professionals during ANC visits.

This study also found that pregnant women who experienced health problems during pregnancies at the moment were six times more likely to use self-medication compared to their counterparts. This indicates that women who have any types of health problem during pregnancy may prefer self-medication to save time and other related factors.

Among women who used self-medication during pregnancies then 20 (40.0%) took during the second trimester, and 15 (30.0%) did so during the first and third trimesters of their pregnancies. However, sufficient evidence was not found about the association between gestational age and self-medication. This is contrary to the finding of study in Ethiopia reported that OTC medications use during pregnancy was observed in the third trimester of pregnancy than in the other trimesters [27].

### Conclusion and recommendations

The prevalence of self-medication among pregnant women in Goba town was comparable to the findings of other studies.

Better maternal education and facing health problems during pregnancies were positively associated with self-medication practices while ANC follow up was found to have a protective factor for self-medication uses. Strengthen existing maternal health services contribute a lot to decrease practices mentioned. Health institutions have to give health education about the risks associated with self-administered drugs to all pregnant women attending ANC services regardless of gestational age and types of health problems; reinforce drug retail outlet’s not to dispense drugs without rational prescriptions, even OTC medication, for pregnant women without considering the risk.

## Limitations

- This study is quantitative; it was better if qualitative approach was also employed to investigate attitudes of study subjects towards self-medication.
- The study has been conducted at the town and results difficulty to generalize for the rural population.

## Additional file

**Additional file 1: Figure S1.** Reasons for self-medication among pregnant women at Goba Town, 2015.

## Abbreviations

ANC: antenatal care
AOR: adjusted odds ratio
CI: confidence interval
EDHS: Ethiopian Demographic Health Survey
IRB: Institutional Review Board
SD: standard deviation
