# Supplementary material for: Self-medication during pregnancy and associated factors among pregnant women in Goba town, southeast Ethiopia: a community based cross sectional study
Source: BMC Res Notes. 2018 Oct 10;11:713. doi: 10.1186/s13104-018-3821-8 (PMC6180449; doi:10.1186/s13104-018-3821-8)
Supplement: Supplementary file 1 — Additional file 1: Figure S1. Reasons for self-medication among pregnant women at Goba Town, 2015. [file 13104_2018_3821_MOESM1_ESM.docx]

Figure S1: Reasons for self-medication among pregnant women at Goba Town, 2015.
